# Formulation and characterization of lamotrigine nasal insert targeted brain for enhanced epilepsy treatment

**DOI:** 10.1080/10717544.2022.2163321

**Published:** 2022-12-29

**Authors:** Rehab Abdelmonem, Hadel A. Abo El-Enin, Ghada Abdelkader, Mohamed Abdel-Hakeem

**Affiliations:** a Department of Industrial Pharmacy, College of Pharmaceutical Sciences and Drug Manufacturing, Misr University for Science and Technology (MUST), 6th of October City, Giza, 12566, Egypt; b Department of Pharmaceutics, National organization of drug Control and Research (NODCAR), Giza, Egypt; c College of Pharmaceutical Science and Drug Manufacturing, Misr University for Science and Technology (MUST), 6th of October City, Giza, Egypt; d Department of pharmaceutical biotechnology, College of biotechnology, Misr University For Science and Technology (MUST), 6th of October City, Giza, Egypt

**Keywords:** Lamotrigine, nasal insert, nano-spanlastics, brain targeting, nano-vesicles, epilepsy

## Abstract

Lamotrigine. (LMT) is a triazine drug has an antiepileptic effect but with low water solubility, dissolution rate and thus therapeutic effect. Spanlastics are nano-vesicular carriers’ act as site-specific drug delivery system. Intranasal route could direct the drug from nose to brain and provide a faster and more specific therapeutic effect. Therefore, this study aimed to upload lamotrigine onto nano-vesicles using spanlastic nasal insert delivery for effective epilepsy treatment via overcoming lamotrigine’s low solubility and improving its bioavailability. Lamtrigine-loaded nano-spanlastic vesicles were prepared by ethanol injection method. To study different formulation factor’s effect on formulations characters; particle size (PS), Zeta potential (ZP), polydispersity index (PDI), entrapment efficiency percentage (EE%) and LMT released amount after 6 h (Q6h); 2^1 and 3^1 full factorial designs were employed. Optimized formula was loaded in lyophilized nasal inserts formulation which were characterized for LMT release and mucoadhesion. Pharmacokinetics studies in plasma and brain were performed on rats to investigate drug targeting efficiency. The optimal nano-spanlastic formulation (F4; containing equal Span 60 amount (100 mg) and edge activator; Tween 80) exhibited nano PS (174.2 nm), high EE% (92.75%), and Q6h > 80%. The prepared nasal inserts (S4) containing 100 mg HPMC has a higher mucoadhesive force (9319.5 dyne/cm^2^) and dissolution rate (> 80% within 10 min) for rapid in vivo bio-distribution. In vivo studies showed considerable improvement brain and plasma’s rate and extent absorption after intranasal administration indicating a high brain targeting efficiency. The results achieved indicate that nano-spanlastic nasal-inserts offer a promising LMT brain targeting in order to maximize its antiepileptic effect.

## 1. Introduction

Lamotrigine (LMT) is a triazine drug that has been approved as an antiepileptic, anticonvulsant, novel thymoleptic, and psychotropic properties (Matsuo, 1999) and is recommended via international guidelines and the neurological disorder association in epilepsy management for improving life quality and avoiding side effects (Goldsmith et al., 2003; Inal et al., 2021). It acts via binding to sodium channels, stabilizing presynaptic neuronal membranes, and inhibiting glutamate release (Betchel & Saadabadi, 2022). Lamotrigine is used in focal onset, primary generalized tonic-clonic, absent, and myoclonic seizures (Allahverdiyev et al., 2018). Lamotrigine is used in oral tablet form, including chewable, dispersible, and disintegrating tablets with first-order kinetics and a t_1/2_ up to 29 h (Betchel & Saadabadi, 2022). It was observed that lamotrigine has a therapeutic dose range higher than 10 mg/L without clinical toxicity (Castel-Branco et al., 2005) and the common oral dosage is 25 mg/day (Mohan et al., 2015). LAM is classified as a class II drug with a pKa equal 5.70. Its solubility in water at 25 °C is 0.17 mg/mL. Therefore, researchers have attempted to increase LAM’s water solubility and dissolution to improve its therapeutic effects (Soleymani et al., 2017).

The science of nanotechnology and nanosystems has been drastically enhanced over the last few decades for its multiple advantages. The unique properties of nanosized drug-delivery systems resulting from small sized particles as well as the large surface area of the vesicles could improve drugs’ solubility, bioavailability, and passive targeting properties (El-Far et al., 2022). Nanoparticles can penetrate cells more easily and efficiently than larger particles, resulting in better drug delivery, particularly when the drugs are generally either loaded on the particles’ surface or involved in their matrix, achieving the highest bioavailability with the fewest side effects by utilizing site-specific drug delivery (Suri et al., 2007).

Many literatures have recommended the use of elastic vesicular nanocarriers or spanlastics (Kipp, 2004; Ould-Ouali et al., 2005; Farghaly et al., 2017). Spanlastics are a novel nano-vesicular carrier system developed by Kakkar and Kaur (Sharma et al. 2020). They are a bilayer cavity consisting of a nonionic surfactant and an edge activator (EA) to improve drug delivery. Spanlastics have many advantages, such as safety, biodegradability, chemical stability, and non-immunogenicity for vesicular carriers (Badria & Mazyed, 2020; Sharma et al., 2020). Spanlastics are used in ocular, oral, topical, nasal, and transungual drug delivery. They are a special class of vesicular carriers that act as site-specific drug delivery (Badria & Mazyed, 2020).

Intranasal route is one of the most effective routes for directly delivering drugs to the brain via olfactory neuron and trigeminal pathway with avoiding hepatic metabolism and bypassingthe blood-brain barrier (BBB) and blood-cerebrospinal fluid barrier (Law et al., 2001; Khan et al., 2016) which could result in dose reduction when compared to oral delivery (Jadhav et al., 2007). Thus, intranasal route is important for drugs used in crisis management such as pain relievers as well as for centrally acting drugs, where the direct pathway from nose to the brain may provide a faster and more specific therapeutic effect (Illum, 2002).

In the present study, we were interested in uploading lamotrigine onto nano-vesicles using nano-spanlastic nasal insert delivery for effective treatment of epilepsy and to overcome lamotrigine’s low solubility, improve its bioavailability, and decrease its side effects by avoiding higher doses. To our knowledge, this could be the first report on using nasal insert as a delivery system for lamotrigine in nano-form using nano-spanlastic vesicles. Some researchers have developed nanoparticles of lamotrigine for intravenous delivery to extend its release only, as Ammar et al., who designed a depot preparation to extend the drug release of lamotrigine for up to 4 weeks (Ammar et al., 2018).

## 2. Materials and methods

### 2.1. Materials

Lamotrigine (LMT) was kindly provided by Global Napi Pharmaceuticals (Giza, Egypt). Span 60 and Tween 80 were purchased from Sigma Aldrich (St. Louis, Missouri, USA). Hydroxypropyl methylcellulose (HPMC) K4M was obtained from Colorcon (Kent, UK). Gelatin, Potassium dihydrogen phosphate, disodium hydrogen phosphate, and glycine were purchased were obtained from El-Nasr Pharmaceutical Chemicals Co. (Cairo, Egypt). Methanol and ethanol HPLC grades were acquired from Sigma Aldrich (St. Louis, Missouri, USA).

### 2.2. Formulation and evaluation of lamotrigine nano-spanlastic vesicles

The ethanol injection method was used to prepare Lamtrigine-loaded nano-spanlastic vesicles dispersions. Lamotrigine (25 mg) and Span 60 were dissolved in ethanol which consider as an organic phase. The organic phase was injected into a preheated aqueous phase (10 mL) in which an edge activator (Tween 80) was previously dissolved at a fixed ratio 1:5 (V/V). Continuous stirring on a magnetic stirrer was performed to allow complete evaporation of ethanol and the subsequent formation of lamotrigine loaded aqueous nanocarrier dispersions. Fine nanovesicle dispersions were promoted by sonication in the ultrasonic water-bath for 5 min (Crest Ultrasonics Corp., Trenton, NJ) (Abdelmonem et al., 2018).

To investigate the Span and edge activator (Tween 80) effect on different response variables, multi level 2^1 and 3^1 full factorial designs were employed using Design-Expert® software (version 7; Stat-Ease, Inc., Minneapolis, MN, USA) as represented in Table 1. Particle size (Y1; PS), Zeta potential (Y2; ZP), polydispersity index (Y3; PDI), entrapment efficiency percentage (Y4; EE%) and the amount of LMT released after 6 h (Y5; Q6h). The design parameters and constraints are represented in Table 1

**Table 1.** Full experimental factorial design parameters’ and constraints’.

Independent variables	Level of variables
**X1**: **Span 60 (mg)**	100, 160 or 200
**X2**: **Tween 80 (mg)**	0 or 100
**Responses**	**Constraints**
**Y1**: Particle size (PS; nm)	Minimize
**Y2**: Zeta potential (ZP; mV)	Maximize
**Y3**: polydispersity index (PDI)	Minimize
**Y4:** Entrapment Efficiency (EE%)	Maximize
**Y5**: The quantity of LMT released after 6 h (Q6h%)	Maximize

### 2.3. Characterization of lamotragine nanocarriers

#### 2.3.1. Particle size (PS), polydispersity index (PDI) and Z-potential analysis (ZP)

PS, PDI and ZP were assessed for the fabricated nano-vesicles by means of a Malvern Zetasizer 2000 (Malvern Instruments Ltd., UK). The measurements were done after suitable dilution (10 folds with de-ionized water) at 25 ± 1 °C (Abdellatif et al., 2017). The ZP assessment was performed by observing the electrophoretic migration of the vesicles in the electrical field. All measurements were carried out in triplicates, and the results were recorded as the mean ± S.D.

#### 2.3.2. Entrapment efficiency percentage (EE%) evaluation and calculation

The nanocarrier dispersion was centrifuged by a cooling centrifuge (Sigma 3 K 30, Germany) at 20,000 rpm for 1 h at 4 °C. Afterward, the sediment was destroyed with methanol and assessed by a UV-Vis spectrophotometer (Shimadzu UV1650 Spectrophotometer, Koyoto, Japan) at 270 nm (Abdellatif et al., 2017). EE% was evaluated by applying the subsequent equation (Abo El-Enin et al., 2022).

(1) EE%=((Entraped drug)/Total drug amount)×100

#### 2.3.3. In-vitro drug release

The in vitro release of lamotrigine from all prepared nano-spanlastic vesicles was evaluated using the dialysis bag technique. Nanovesicles dispersion containing LMT equivalent to 5 mg was accurately weighed and transferred to a cellulose dialysis membrane having a molecular weight cutoff of 12,000 to 14,000 Da. The membrane was immersed in 150 mL of 10% ethanolic phosphate buffer at pH 7.4 (to maintain sink conditions), the temperature was monitored at 37 °C ± 0.5, and the mixture was stirred at 50 rpm. For 6 h, equal volume samples were drawn at regular time intervals, and the dissolution medium was replaced with a preheated, fresh one. The cumulative LMT amount released was determined spectrophotometrically at 270 nm. The same method was repeated with the drug-free nano-spanlastic vesicles to be used as a blank. All measurements were done in triplicate, and the results were represented as mean ± SD.

#### 2.3.4. Optimization of LMT-loaded s nano-panlastic vesicles

Following the analysis of all responses, Design-Expert® software was used to express the relation between dependent (responses) and independent variables (factors) to determine the optimized formulations according to the required constraints (goals).

#### 2.3.5. Transmission electron microscopy (TEM)

The optimum nanovesicles’ morphology was observed via TEM (Joel JEM 1230, Tokyo, Japan). One drop of the optimum nano-carrier (without dilution) was arranged as a thin film on a carbon laminated copper grid, stained employing phosphotungstic acid 1.5% (Abo El-Enin et al., 2022).

### 2.4. Preparation of lamotrigine nano-spanlastics nasal inserts

The selected prepared lamotrigine nano-spanlastic vesicles formulation was used in preparation of the nano-spanlastics nasal inserts. The nano-spanlastics nasal inserts were prepared according to a 2^3^ full factorial design. Three factors (polymer type, polymer amount) and the collapse agent concentration (Glycine) at two levels actual statistical design was employed as shown in Table 2 using Design Expert 12.0.3.

**Table 2.** The composition of different prepared lamotrigine nano-spanlastics nasal inserts formulations.

#F	Matrix former		Glycine amount (mg)
	Type of polymer	Polymer amount (mg)	
**S1**	HPMC	200 mg	50 mg
**S2**	HPMC	100 mg	100 mg
**S3**	HPMC	200 mg	100 mg
**S4**	HPMC	100 mg	50 mg
**S5**	Gelatin	200 mg	50 mg
**S6**	Gelatin	100 mg	100 mg
**S7**	Gelatin	100 mg	50 mg
**S8**	Gelatin	200 mg	100 mg

Briefly, a sufficient amount of the selected matrix former polymer (1% w/w gelatin or 2% w/w HPMC) and the required amount of glycine (collapse protecting agent) as represented in Table 2 were mixed with mannitol (20 g) as an insert filler and sweeting agent. All were dissolved in one third of the required amount of distilled water (Table 2). The selected nano-spanlastic dispersion formula with an equivalent lamotrigine amount (2 mL) was mixed with the previously prepared aqueous solution (8 mL) and stirred for 10 min using a magnetic stirrer at 500 rpm at 37 °C) till homogeneity (El Nabarawi et al. 2016).

One milliliter of the prepared suspension was then poured into the polypropylene tubes as a mold. The tubes were lyophilized after being frozen at −22 °C for 24 h to prepare the lyophilized inserts. The frozen inserts were then placed in the lyophilizer (Freeze dryer; LABCONCO-USA) for 24 h in a freeze-dryer with the preset cycle stages; freeze for 4 h at −30 °C, dry for 20 h with vacuum 50 m Torr with condenser temperature at −50 °C throughout the process. The lyophilized inserts were removed from the used mold (polypropylene tubes) and kept in tightly closed containers in desiccators over anhydrous calcium chloride (29% relative humidity) at room temperature till further use. Each insert was tested for its appearance and physical properties, including shape, color, and texture.

### 2.5. Evaluation of the prepared nasal insert

#### 2.5.1. Determination of drug content of nasal insert

One insert from each formulation was dissolved in100 mL ethanol using an Incubator Shaker (JEIO Tech Si-300, kyonggi-Do, Korea) at 37 °C. Aliquots were withdrawn and filtered and the average drug content in triplicate manner for each formula was determined spectrophotometrically at 270 nm after suitable dilution.

#### 2.5.2. Friability

One insert from each formula was accurately weighted and placed in the drum of a friabilator, which rotated at 25 rpm for a period of 4 min. The inserts were then brushed and reweighed. The percentage loss in weight was calculated and taken as a measure of friability.

#### 2.5.3. Wetting time

Ten milliliters of distilled water containing eosin, a water-soluble dye, were placed in a Petri dish of 10 cm diameter (El-Nabarawi et al., 2013). The insert was carefully placed in the center of the Petri dish, and the time required for the dye to reach the upper surface of the insert was noted as the ‘wetting time’.

#### 2.5.4. Determination of surface pH

Agar solution was prepared by dissolving 2% w/v agar in distilled water and heating it with stirring, before pouring it into petri dish to solidify at the room temperature. The plain inserts were left to swell for 2 h on the surface of an agar plate. Surface pH was measured by means of a pH paper (Whatman full range 1–14) placed on the surface of the swollen inserts (Seager, 1998).

#### 2.5.5. Disintegration time

The inserts was placed in 10 mL phosphate buffer solution, pH 7.4 at 37 ± 0.5 °C. The time required for complete dispersion of insert was recorded as the in-vitro disintegration time (DT).

### 2.6. Determination of lamotrigine released from different prepared nasal insert formulae

The study was carried out using a modified USP dissolution apparatus II. A 10 mL capacity syringe was prepared to act as a tube by smoothly cutting the whole diameter near the nozzle (El-Hadidy, 2010). Each nasal insert formula was introduced into the syringe from the top after removing the pump. The syringe was covered upside with cellulose membrane and filled with 2 mL phosphate buffer pH 7.4 before being attached to the rotating paddle. The syringe tube was immersed in the vessel containing 200 mL distilled water at 37 C ± 0.5 °C with a paddle speed of 50 rpm. Aliquot of (2 mL) was withdrawn at specified regular time interval over 60 min and immediately replaced with fresh release medium. The drug content in the withdrawn samples was determined spectrophotometrically at 270 nm (UV spectrophotometer; UV-1650 P.C Shimadzu, Japan).

### 2.7. Determination of mucodhesion of lamotrigine inserts formulae

The experimental technique for determining bioadhesive force was derived from a previously described method that was reported by Shivhare et al. (2009) The apparatus was developed to determine the minimum weight required for separating two membranes from each other with a polymer film spread between them, as described in Yong et al. (2001) and El-Nabarawi et al. (2012).

### 2.8. In-vivo studies of the selected formula

To investigate the effectiveness of the LMT nasal insert in improving the LMT’s brain targeting, a parallel design was employed. Seventy-two male Wister albino rats (200–250 g) were divided into three groups, each containing 24 rats. Group 1 received oral lamotrigine powder suspended in distilled water; group 2 received a freeze dried nasal insert (S4) loaded with lamotrigine suspension; while group 3 received the selected freeze dried nasal insert formula (S4) loaded with the selected lamotrigine nano-spanlastic vesicles formula (F4). All rats were administered the same LMT amount dose (25 mg/250 g) (Abdelmonem et al., 2020). Nasal inserts and nano-spanlastic nasal insert (approximately 0.7 cm) were instilled into rats’ nostrils with the help of a microinjector equipped with a soft polyethylene tube having 0.10 mm internal diameter at the delivery site (Musumeci et al., 2018). The experiments were done according to the ARRIVE guidelines and approved by the Must research ethical committee, FWA00025577, Must University, Egypt.

At different time intervals 1/2, 1, 2, 4, 6, 8, 10 and 24 h following lamotrigine administration, rats were scarified after were anesthetized by diethyl ether inhalation. Plasma samples were separated from the blood, which was collected from the trunk then placed into heparinized tubes after being centrifuged at 4000 rpm for 15 min. Brain tissue samples were taken at the same time intervals, just after blood collection. The skulls were cut, opened, and the collected brains were homogenized with three folds brains’ volumes with distilled water at 24,000 rpm for 1 min (Fukami et al., 2005; Costantino et al., 2007). Homogenized brain and separated plasma were stored at −80 °C. Lamotrigine levels in plasma and brain homogenate were determined using a high-performance liquid chromatography (HPLC) method previously described by Castel-Branco et al., 2001 (Castel-Branco et al., 2003). The UV detector was set at 270 nm.

Pharmacokinetic parameters; maximum plasma concentration (C_max_), time at peak plasma concentration (T_max_), area under plasma concentration during evaluation time (24 h); (AUC_0-24_), mean residence time (MRT) and half-life (t_1/2_) were calculated using the pharmacokinetic software PK Solver-Add Ins for Microsoft Excel 2007.

### 2.9. Statistical analysis

All data were expressed as the mean ± standard deviation (SD) of three replicates. Design-Expert 7® Software, version 7, Stat-Ease, Inc., Minneapolis, MN, USA, was used for formulation design and evaluation. One-way ANOVA was used to evaluate the effect of formulation factors on the selected formulations’ characteristics, with *p* > 0.05 considered statistically significant. One-way Analysis of Variance (ANOVA) was applied to define the formulation effect on the tested pharmacokinetic parameters, considering that the *p* value < 0.0001 is a significant effect, followed by the Tukey-Kramer test and Guassian’s test for multiple comparisons and to determine the source of difference, respectively.

## 3. Results and discussion

### 3.1. Preparation and characterization of lamotragine nano-spanlastics vesicles

The main goal for brain targeting nasal insert preparation is obtaining uniform nano-size vesicles with maximum stability, and the highest entrapment efficiency additionally improving the drug dissolution rate for improving the nasal drug entrapping. Using Tween 80 as an edge activator with its hydrophilic characters improves the nano-spanlastics vesicles elasticity to squeeze themselves easily through the nasal mucosa pores layer. Span 60 is lipophilic, nonionic surfactant helps in improving LMT poor solubility and promotes the formation of mono and/or multi-lamellar nano-vesicles (Fahmy et al., 2018).

Table 3 shows the different prepared formulae composition and their effect on the PS, ZP, PDI, EE%, and Y5: The quantity of LMT released after 6hs (Q6h%) results from six LMT- nano-spanlastics formulations. All factors have a significant effect on the tested responses, and follow a selected two factor interaction model with R2 value > 0.998. Statistical analysis results are shown in Table 4. The high R^2^ ensures the closeness of predicted and experimental results as well as the ability of the developed model to predict the experimental results. The signal-to-noise ratio was more than 4, indicates adequate precision, which means the models’ ability to Span the design space (Leng et al., 2018). The final equations in terms of coded factors are represented in Table 5.

**Table 3.** Experimental runs, the composition and the measured responses for the prepared different prepared LMT formulations.

Runs	Factors		Responses				
	X1 Span 60 (mg)	X2 Tween 80 (mg)	**Y1**: (PS; mm)	**Y2**: (ZP; mV)	**Y3**: (PDI)	**Y4:** (EE%)	**Y5**: (Q6h%)
F1	100	0	301.6 ± 5.22	−20.5 ± 0.8	0.349 ± 0.08	98%±2.65	62.58%±2.45
F2	160	0	253.9 ± 2.32	−38.5 ± 0.84	0.429 ± 0.02	97.00%±3.70	62.78%±3.7
F3	200	0	190 ± 7.40	−40.1 ± 1.5	0.599 ± 0.1	48.50%±5.00	54.24%±2.01
F4	100	100	174.4 ± 2.11	−39.7 ± 0.75	0.566 ± 0.02	93%±2.00	80.88%±2.31
F5	160	100	255.2 ± 3.50	−32 ± 1.56	0.629 ± 0.05	63%±3.07	66.15%±3.04
F6	200	100	274.5 ± 3.21	−32.6 ± 0.87	0.478 ± 0.03	75%±3.50	65.97%±3.7

PS: Particle size, ZP: Zeta potential, PDI: polydispersity index, EE%**:** Entrapment Efficiency and Q6h%: The quantity of LMT released after 6 h.

**Table 4.** The design expert results of all response variables.

Source	PS (nm)		ZP		PDI		EE%		Q6h%	
	*F*	*p*-value	*F*	*p*-value	*F*	*p*-value	*F*	*p*-value	*F*	*p*-value
Model	22,368.39	< 0.0001	833.60	<0.0001	803.71	<0.0001	7771.54	<0.0001	685.8739	<0.0001
A-Span 60 (mg)	2471.30	< 0.0001	343.1615	<0.0001	254.4694	<0.0001	10,622.01	<0.0001	625.5556	<0.0001
B-Tween 80 (mg)	2528.99	< 0.0001	58.44099	<0.0003	1004.574	<0.0001	496.1938	<0.0001	1890.059	<0.0001
*AB*	52,185.19	< 0.0001	11711.609	<0.0001	1252.516	<0.0001	8558.752	<0.0001	144.0998	<0.0001
Mean	241.3		−33.6583		0.504917		78.85833		64.635	
Adequate Precision	384.68		76.44655		72.87527		213.5089		77.44806	
R^2^	0.9999		0.998563		0.998509		0.999846		0.998253	
Adjusted R^2^	0.9999		0.997365		0.997267		0.999717		0.996798	
Predicted R^2^	0.9998		0.99425		0.994037		0.999382		0.993014	
SD	0.4673		0.366288		0.005346		0.327872		0.480607	
%CV	0.1936		1.088253		1.058856		0.415773		0.743571	

**Table 5.** Final equation in terms of the tested factors.

	PS (nm)	ZP	PDI	EE%	Q6h%
**Intercept**	+241.30	−33.66	+0.50	+78.86	+64.64
A-Span 60 (mg)	12.975	3.86	−0.049	+16.39	+6.73
B-Tween 80 (mg)	−6.78	−0.81	+0.049	−2.01	+6.03
*AB*	−56.77	−8.74	+0.058	−0.39	+3.04

PS: Particle size, ZP: Zeta potential, PDI: polydispersity index, EE%**:** Entrapment Efficiency and Q6h%: The quantity of LMT released after 6 h.

**Figure 1.**
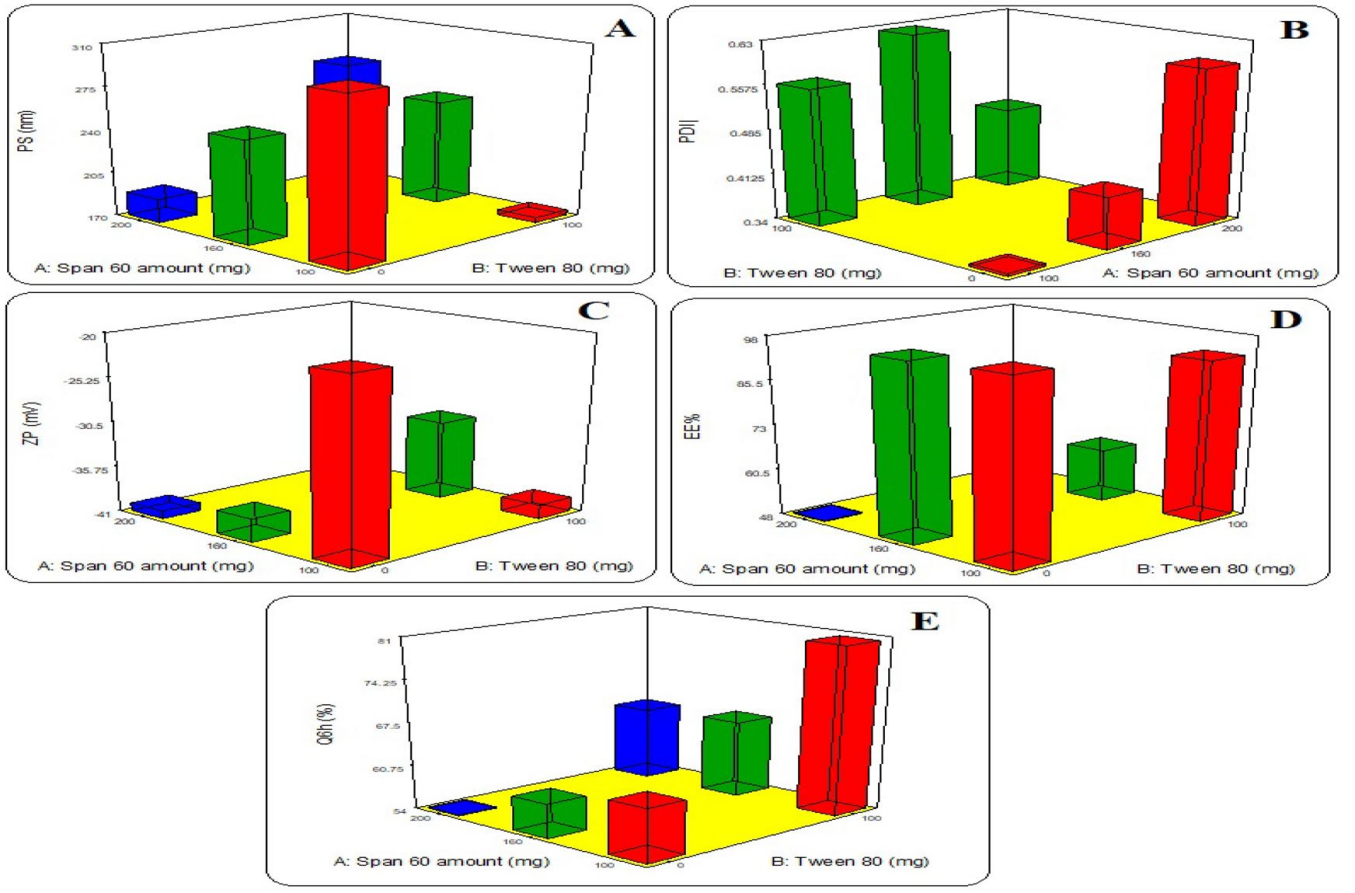
Effects of formulation variables on (A) Particle size (PS), (B) Zeta potential (ZP), (C) polydispersity index (PDI), (D) Entrapment Efficiency (EE%) and (E) The quantity of LMT released after 6 h (Q6h%).

All formulations have a nano particle size ranging from 174.4 nm to 301.6 nm. As represented in Table 4 and Figure 1(A), increasing the used Tween 80 (HLB; 15) concentration led to a significant decrease in the spanlanstic vesicles size while increasing the Span 60 (HLB; 4.6) concentration increases the vesicles’ size. This was in agreement with previously reported by Konaiko et al., 2015, who reported that there was an inverse relation between PS and HLB value (Komaiko & McClements, 2015). Tween 80 as edge activator could condense nano-spanlastic vesicles membranes via enhancing those reorders. For Span 60; the increase in the PS was related to increasing the incorporation of more alkyl chains through the hydrophobic region of vesicles, which increases the incorporated drug in the vesicles (Tayel et al. 2015). To be more precise, Span 60 at a concentration greater than 160 mg led to decreasing the vesicles’ size. This might be because of interfacial stress reduction with high EA concentration as reported by Shamma et al. (2019). According to the expressed equation (Table 5 and Figure 1(A)), it was found that all factors have significant negative effect on the PS, leading to the formation of a nano sized spanlastics vesicles’.

The PDI as shown in Table 4 and Figure 1(B), ranged from 0.349 to 0.629. The polydispersity index (PDI) represents the degree of any colloidal system homogeneity by indexing the size of the formed nanoparticle system distribution (El-Far et al., 2022). The PDI value should be less than 0.7 as the smaller PDI is the narrower particles’ size distribution and forming a homogenous, monodisperse system (Abdellatif et al., 2019).

The surface charge potential is indicated by the zeta potential value, which maintains the physical stability of the nano-spanlatics vesicles by reducing the aggregation possibilities of the formulation by increasing the repulsion force between the particles, which can overcome the van der Waals attractive forces and thus prevent particle aggregation (Alzubaidi et al., 2017) especially for the nanosystem with a Z-potential value greater than ±30 (Aziz et al., 2018). The prepared nano-spanlastic vesicles have accepted negative Z-potential values that ranged from − 20.5 ± 0.8to − 40.1 ± 1.5 mV (Table 3). The hydroxyl groups of the used nonionic surfactants cause the vesicles’ negative charge (Junyaprasert et al., 2008). Span amount has a significant effect on the zeta potential value, while increasing the Tween 80 amount produces an insignificant effect (Table 3 and Figure 1(C)). There was a significant interaction between the used surfactants in formula F4, as increasing the surfactants amounts (Span 60 or Tween 80) increases the negative zeta potential value. This was related to the stronger dipole and the higher polarity of the used surfactants (Restuinjaya et al., 2019).

The polynomial equation of the EE% (Table 5) demonstrates that the relation between EE and independent formulation factors is a positive effect in related to increasing Span 60 amounts while it was in a negative effect with increasing the Tween 80 amount. The combined effect of both surfactants led to decreasing the EE% (Figure 1(D)). Therefore, it was recommended to increase the Span 60 with decreasing the Tween 80 amount. The later could be related to the Span 60’s hydrophobicity and its high phase transition temperature; 53 °C (Yoshioka et al., 1994). Besides the high HLB value of Tween 80 increases its hydrophilicity, which could increase the nano-spanlastic visceles’ permeability and the probability of drug leakage (Fahmy et al., 2019).

**Figure 2.**
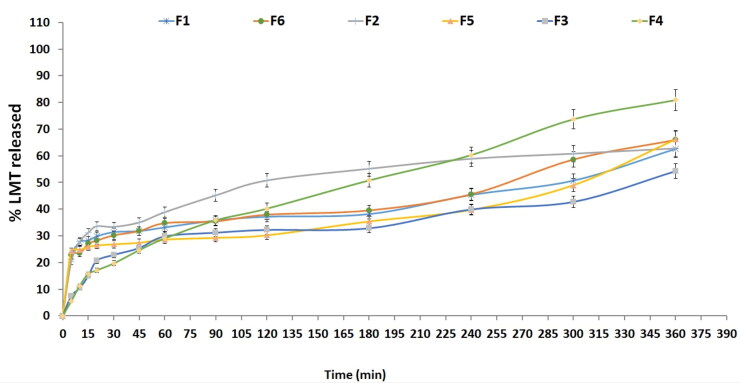
Release profile of different nano-spanlastic vesicles’ formulations.

Figure 2 represents the release profile of LMT from different nano-spanlastic formulations have a biphasic release profiles. The first is a quick release rate, more than 30% in the initial 90 min (except for formula F5), which due to releasing the uncapsulated LMT, while the other release profile is a sustained release profile up to 6 h. There was a significant effect of the edge activator on the LMT release profile (Table 4). Increasing Tween 80 amount led to increasing LMT amount released. This result is in agreement with previously reported by De Jesús Valle and Zarzuelo Castañeda et al. who found that the drug release could be explained by the difference in molecular packing and deformability associated with the edge activator used (De Jesús Valle et al., 2022). The presence of Tween 80 at high concentration could improve the drug’s solubility by raising nano-spanlastic bilayer fluidity, resulting in an improvement of membrane permeability and hence, forward improving the release rate (Al-Mahallawi et al., 2014). On the other hand, increasing the Span 60 amount significantly decreases LMT release rate. HLB value of Span 60 is slightly lipophilic (Dehghan & Marzuka, 2014). This could be due to LMT’s lipophilic nature, which prefers to be entrapped in relatively lipophilic spanlastics rather than dispersed in the polar external release media (Bhunchu et al., 2015). As shown in Table 5 and Figure 1(E); the significant combined effect of the used surfactant (Span 60) and the edge activator (Tween 80) increases the LMT release profile as in formula F4. This could be due to decreasing particle size, as smaller particles have a larger surface area, which improves drug release (El Menshawe et al., 2019).

### 3.2. Optimization of LMT nano spanlastic vesicles

By studying the responses, the optimum levels of the variables were predicted. The calculated desirability value was 0.7198. Characterization was performed on the optimized formulation that had been prepared. There were no significant residual errors found, indicating that numerical optimization was appropriate for this study. The results indicated that the optimized formula shows nanoparticle size 174.2 nm, EE 92.75%, ZP −39.35 mV, PDI value =0.563 and Q6h up to 80.44%. These results indicated that the optimized LMT nano-spanlastic formula No. 4 (F4) is the best selected formula.

### 3.3. Tem morphological evaluation of the selected nano-spanlastic LMT

**Figure 3.**
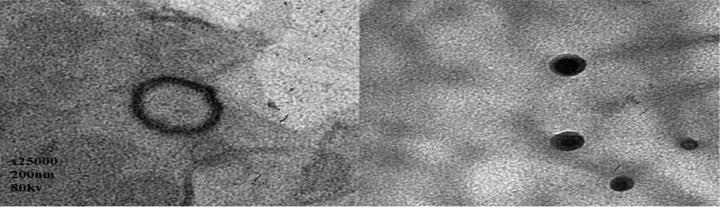
Transmission electron microscope photography of LMT nano-spanlastic vesicles formula F4 (×25,000).

Figure 3 demonstrates the transmission electron microscope examination of the optimized LMT loaded nano-spanlastic vesicles. The TEM photographs depicted that almost spanlastic vesicles were spherical in shape with nanosize unilamellar visceles. They have no aggregation or drug crystals. The vesicles have a defined boundaries, as the used nonionic surfactants with its amphiphilic nature tend to develop spherical vesicles to decrease the surface-free energy (Mazyed et al., 2021).

### 3.4. Preparation of lamotrigine nano-spanlastics nasal inserts

Nasal inserts were prepared using 2^^3^ factorial design (Table 2). Mannitol was added as an insert filler and sweetening agent to avoid the bitter taste of postnasal secretion. All the prepared lyophilized nasal inserts have a white color, with a spongy appearance and a smooth surface. The porous structure is an ideal for rehydration when placed in the nose with minimal discomfort for the nasal cavity (Alfadhel et al., 2011).

### 3.5. Physical evaluation of lamotrigine nano-spanlastics nasal inserts

Table 6 represented the physical characteristic of the prepared lamotrigine nano-spanlastics nasal inserts. All the prepared nano-spanlastic nasal inserts have LMT amounts more than 80%. The drug content was ranged from 84.92% ±1.23 to 101.37% ±3.57 indicating uniformly drug distributed in the polymeric matrix. The later proves the adopted of preparation method in producing uniform inserts (Dae-Duk, 2007). The prepared inserts did not show any capping or breaking and had an accepted friability test (less than 1%) except for formula S5 (1.09±.11). Increasing glycine concentration as a collapse protectant agent has an inverse effect on insert friability and wetting ability. HPMC improves the inserts’ wetting ability and reduces the average wetting time of different formulations compared to those prepared using gelatin. Therefore formula S8 has the highest wetting time related to high glycine (100 mg) concentration and gelatin (200 mg) concentration. The pH value of all formulations is acceptable, as all are ranged from 6 to 6.5 except formulae S2, S6 and S8. They are slightly shifted from the accepted range. The pH of the prepared formulations should be close to the nasal mucosa’s (5.0 to 6.5) to avoid causing sensations of discomfort, irritation or toxicity in the nasal epithelium and/or enhanced mucociliary clearance (Pires et al., 2022). The mucoadhesion force of the prepared nasal inserts was ranged from 7063.2 ± 2.06 to 9319.5 ± 2.02 dyne/cm2 (Table 6). The mucoadhesion force of HPMC type nasal insert S1:S4 was greater than the mucoadhesion force of gelatin type nasal insert S5:S8.

**Table 6.** Physical evaluation of lamotrigine nano-spanlastics nasal inserts.

Samples	Drug content (%)	Friability (%)	Wetting time (sec)	Disintegration time (sec)	pH	**Mucoadhesive test** **(dyne/cm^2^)**
**S1**	86.29 ± 1.28	0.60 ± 0.12	4 sec	30 ± 4.00	6.5 ± 0.50	9025.2 ± 2.03
**S2**	85.85 ± 2.34	1.00 ± 0.10	6 sec	30 ± 2.00	6.7 ± 0.20	7946.1 ± 2.04
**S3**	87.28 ± 2.34	0.86 ± 0.12	3 sec	30 ± 1.00	5.6 ± 0.40	7749.9 ± 3.01
**S4**	101.37 ± 3.57	0.11 ± 0.09	2 sec	15 ± 2.00	6.0 ± 0.20	9319.5 ± 2.02
**S5**	86.27 ± 2.65	1.09±.11	7 sec	120 ± 1.00	5.8 ± 0.12	8632.8 ± 2.01
**S6**	85.81 ± 3.45	0.58 ± 0.12	10 sec	60 ± 3.00	6.6 ± 0.20	7259.4 ± 3.02
**S7**	85.35 ± 1.89	0.47 ± 0.13	9 sec	60 ± 5.00	6.5 ± 0.40	7063.2 ± 2.06
**S8**	84.92 ± 1.23	0.90 ± 0.10	15 sec	120 ± 5.00	6.8 ± 1.20	7749.9 ± 2.03

### 3.6. In vitro drug release profile from different LMT nano-spanlastics nasal inserts formulae

**Figure 4.**
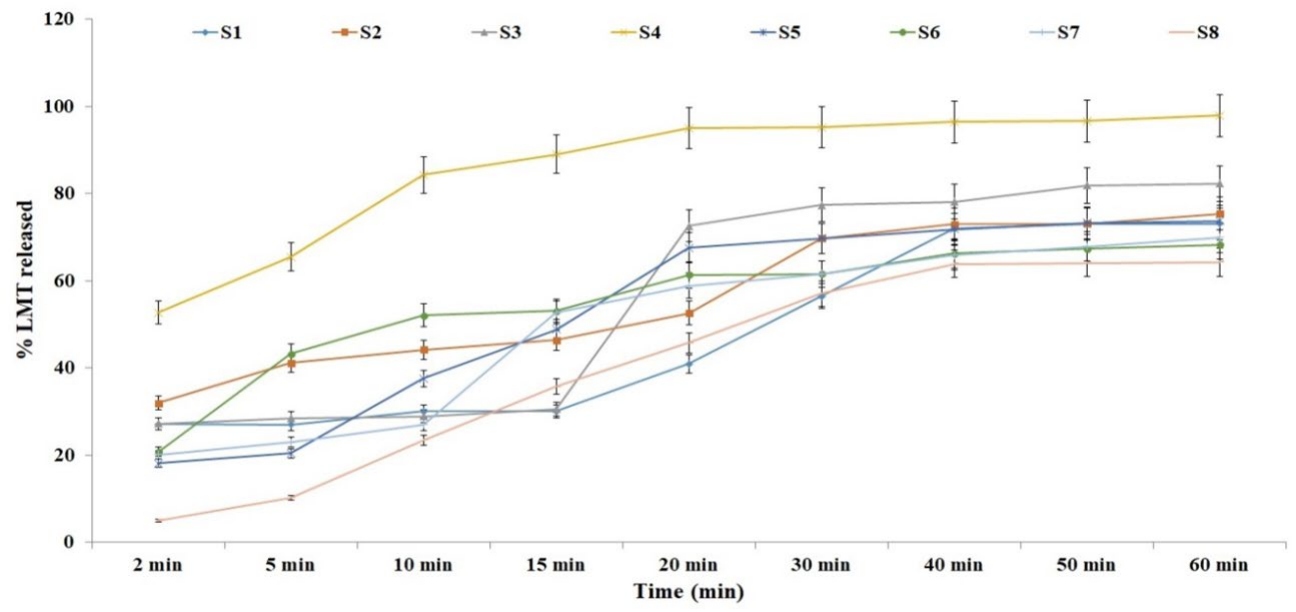
*In vitro* LMT nasal inserts release.

The release rate was studied for 60 min taking into consideration the eventual mucocillary clearance and the limited nasal residence. Figure 4 illustrates the cumulative LMT released from different nasal inserts. The release rate of LMT from freeez dried spanlastic nasal insert was higher than that noticed from the prepared selected nano-spanlastic vesicles. This was due to the lyophilized formula being reasonably rapidly hydrated, which facilitated the drug release. It had a different release rate according to the amount and the type of the polymer used. The fastest release rate was observed from formula S4 as more than 80% of the LMT amount was released in the first 10 min. On the other hand, formula S8 had a slower rate than any other formula as it contained the highest amount of the polymer used and the highest glycine concentration. Using HPMC improves the release rate (S1:S4) when compared to the other formulae that have gelatin at the same concentration (S5:S8). The release results were also related to the wetting test. This was consistent with previous findings that drug release from nasal inserts is related to water penetration and the polymer’s ability to relax, swell, and spread within the inserts (Bertram & Bodmeier, 2006). Using a nano system in the insert could enhance the polymer and drug solubility and hence its dissolution and diffusion through the rehydrated porous lyophilized inserts (Dehghan & Marzuka, 2014). From the previously obtained physical evaluation results and the in vitro release result, formula S4 was selected for further studies.

### 3.7. In vivo study of lamotrigine nano-spanlastics nasal inserts

**Figure 5.**
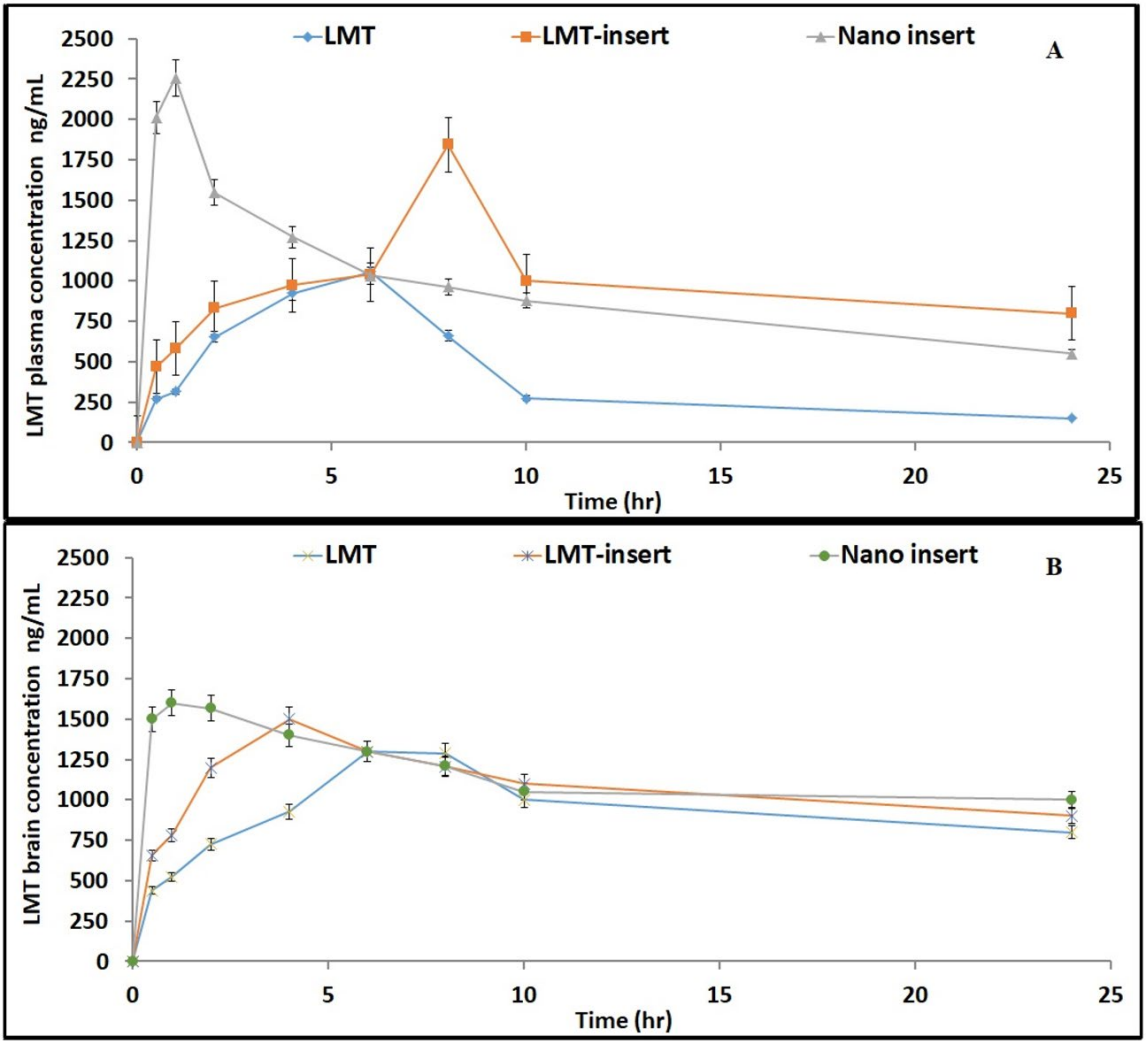
LMT concentrations in rats after administration of various formulations: (A) Plasma concentrations, (B) Brain concentrations.

The mean plasma levels after administration of the different LMT treatments versus time are presented in Figure 5 and the different pharmacokinetic parameters are presented in Table 7.

**Table 7.** Calculated pharmacokinetic parameters following administration of different LMT formulations.

	Group 1 Lamotrigine powder suspended (Peroral)		Group 2 Lamotrigine loaded in the selected freeze dried nasal insert formula (Intranasal)		Group 3 Freeze dried selected Lamotrigine nano-spanlastic vesicles (Intranasal)	
Pharmacokinetic parameter	Plasma	Brain	Plasma	Brain	Plasma	Brain
T_max_ (hr)	6.00	6.00	4.00	4.00	1.00	1.00
C_max_ (ng mL^−1^)	1058.33	1300	1638.5	1500	2258.3	1600
AUC_0-24_ (ng hr mL^−1^)	9885.76	22,335.3	16,626.1	25,823.8	22,440.8	27,520
T_1/2_ (hr)	8.24687	28.1307	10.2955	37.2903	20.1331	78.9839
MRT (hr)	12.2245	42.8463	14.9787	54.6141	27.4502	113.102

All results are recorded as mean ± SD, *n* = 6.

It was noticed that nasal administration of different LMT formulations significantly has higher C_max_, AUC_(0–24)_ and shorter T_max_ than oral LMT suspension. Nano-spanlastic formula significally increases drug concentration in brain and plasma after nasal administration than any other formulae. The AUC_(0–24)_ after administration of nano-spanlastic lyophilized formula 2.2 times that of drug suspension administered orally. . The increased drug concentration in the brain following LMT-nano-spanlastic formula administration could be attributed to the nano-vesicle size, which allows drug particles to be transported deeper into the olfactory epithelial cell layers, overcomes the BBB, and has a higher cellular uptake (Gao & Jiang, 2006; Acosta, 2009; Mistry et al., 2015). This was observed when comparing the nasal administration of nano-spanlastic formula with freeze dried LMT administration. The shorter T_max_ in the brain than in plasma after LMT-nano-spanlastic administration indicated the rapid passage and targeting of the drug to the brain. Additionally, a significant increase in the AUC_0–24_ results suggests that intranasal nano-spanlastic LMT enhances the drug rate and extent absorption. The LMT plasma concentrations of nano-spanlastic formula S4 and freeze dried LMT formulations demonstrated a double-peak phenomenon, which could be caused by the secondary release of the in-vivo swollen part of LMT. The plasma concentration of the swollen LMT from formulation nano-spanlastic formula S4 still exceeds the freeze dried LMT. The spongy structure approves their absorption than oral one.

Finally, it was discovered that administering LMT intra-nasally as freeze-dried nano-spanlastic vesicles (S4) increased the drug’s bioavailability by shortening the time required to reach peak plasma concentration, increasing the extent of absorption, and increasing its ability to cross the BBB, resulting in superior pharmacokinetic profiles.

## 4. Conclusion

Nano-spanlastic LMT could be successfully prepared using an equal amount (100 mg) of Span 60, and the edge activator (Tween 80). This formula could significantly improve LMT release rate (> 80% after 6 h) and has a high EE% (92.75%), with a small PS, a uniform spherical shape (174.2 nm), and high stability, as represented by its high negative zeta potential value (−39.35). Optimum LMT nano-spanlastic vesicles could be formulated in intranasal inserts after freeze drying. HPMC as a polymer at 100 mg has a higher mucoadhesive force (9319.5 ± 2.02 dyne/cm^2^) than using gelatin as a polymer and produces inserts that did not show any capping or breaking. Using glycine as a collapse protecting agent (50 mg) reduced the formulation’s friability (0.11 ± 0.09%). The prepared nasal inserts (S4) have the lowest wetting time (2 sec) and disintegration time (15 ± 2.00 sec) for rapid in vivo biodistribution. Using a nano system in the insert could enhance the drug’s solubility and dissolution rate. The in vivo study suggests that intranasal nano-spanlastic LMT enhances the drug’s absorption rate and extent when targeting the drug to the brain. Based on these findings, nano-spanlastic nasal inserts could be regarded as a novel innovative nano-carrier delivery system for brain targeting of LMT in order to maximize its therapeutic effect.

## Supplementary Material

Supplemental Material
