# Supplementary material for: Formulation and characterization of lamotrigine nasal insert targeted brain for enhanced epilepsy treatment
Source: Drug Deliv. 2022 Dec 29;30(1):2163321. doi: 10.1080/10717544.2022.2163321 (PMC9809415; doi:10.1080/10717544.2022.2163321)
Supplement: Supplemental Material [file IDRD_A_2163321_SM9217.docx]

**Supplementary Data**

One-way Analysis of Variance (ANOVA)

The P value is < 0.0001, considered extremely significant.

Variation among column means is significantly greater than expected by chance.

Tukey-Kramer Multiple Comparisons Test

If the value of q is greater than 4.339 then the P value is less

than 0.05.

Mean

Comparison Difference q P value

================================== ========== ======= ===========

Column A vs Column B -6729.3 343.35 *** P<0.001

Column A vs Column C -12547 640.20 *** P<0.001

Column B vs Column C -5817.9 296.85 *** P<0.001

Mean 95% Confidence Interval

Difference Difference From To

================================== ========== ======= =======

Column A - Column B -6729.3 -6814.3 -6644.3

Column A - Column C -12547 -12632 -12462

Column B - Column C -5817.9 -5902.9 -5732.8

ANOVA assumes that the data are sampled from populations that follow

Gaussian distributions. This assumption is tested using the method

Kolmogorov and Smirnov:

Group KS P Value Passed normality test?

=============== ====== ======== =======================

Column A Too few values to test.

Column B Too few values to test.

Column C Too few values to test.

Intermediate calculations. ANOVA table

Source of Degrees of Sum of Mean

variation freedom squares square

============================ ========== ======== ========

Treatments (between columns) 2 2.366E+08 1.182E+08

Residuals (within columns) 6 6914.0 1152.3

---------------------------- ---------- --------

Total 8 2.366E+08

F = 102645 =(MStreatment/MSresidual)

Summary of Data

Number Standard

of Standard Error of

Group Points Mean Deviation Mean Median

=============== ====== ======== ========= ======== ========

Column A 3 9920.2 48.297 27.884 Unknown

Column B 3 16650 22.220 12.829 Unknown

Column C 3 22467 25.113 14.499 Unknown

95% Confidence Interval

Group Minimum Maximum From To

=============== ======== ======== ========== ==========

Column A Unknown Unknown 9800.3 10040

Column B Unknown Unknown 16594 16705

Column C Unknown Unknown 22405 22530

* * *

**Test Cmax plasma**

One-way Analysis of Variance (ANOVA)

The P value is < 0.0001, considered extremely significant.

Variation among column means is significantly greater than expected by chance.

Tukey-Kramer Multiple Comparisons Test

If the value of q is greater than 4.339 then the P value is less than 0.05.

Mean

Comparison Difference q P value

================================== ========== ======= ===========

Column A vs Column B -573.32 69.895 *** P<0.001

Column A vs Column C -1204.8 146.88 *** P<0.001

Column B vs Column C -631.47 76.985 *** P<0.001

Mean 95% Confidence Interval

Difference Difference From To

================================== ========== ======= =======

Column A - Column B -573.32 -608.91 -537.73

Column A - Column C -1204.8 -1240.4 -1169.2

Column B - Column C -631.47 -667.06 -595.88

ANOVA assumes that the data are sampled from populations that follow

Gaussian distributions. This assumption is tested using the method

Kolmogorov and Smirnov:

Group KS P Value Passed normality test?

=============== ====== ======== =======================

Column A Too few values to test.

Column B Too few values to test.

Column C Too few values to test.

Intermediate calculations. ANOVA table

Source of Degrees of Sum of Mean

variation freedom squares square

============================ ========== ======== ========

Treatments (between columns) 2 2178945 1089473

Residuals (within columns) 6 1211.1 201.84

---------------------------- ---------- --------

Total 8 2180156

F = 5397.6 =(MStreatment/MSresidual)

Summary of Data

Number Standard

of Standard Error of

Group Points Mean Deviation Mean Median

=============== ====== ======== ========= ======== ========

Column A 3 1073.1 16.293 9.407 Unknown

Column B 3 1646.4 6.851 3.955 Unknown

Column C 3 2277.9 17.121 9.885 Unknown

95% Confidence Interval

Group Minimum Maximum From To

=============== ======== ======== ========== ==========

Column A Unknown Unknown 1032.6 1113.6

Column B Unknown Unknown 1629.4 1663.4

Column C Unknown Unknown 2235.3 2320.4

* * *

**Test Cmax brain**

One-way Analysis of Variance (ANOVA)

The P value is < 0.0001, considered extremely significant.

Variation among column means is significantly greater than expected by chance.

Tukey-Kramer Multiple Comparisons Test

If the value of q is greater than 4.339 then the P value is less than 0.05.

Mean

Comparison Difference q P value

================================== ========== ======= ===========

Column A vs Column B -203.33 15.578 *** P<0.001

Column A vs Column C -313.33 24.005 *** P<0.001

Column B vs Column C -110.00 8.427 ** P<0.01

Mean 95% Confidence Interval

Difference Difference From To

================================== ========== ======= =======

Column A - Column B -203.33 -259.97 -146.70

Column A - Column C -313.33 -369.97 -256.70

Column B - Column C -110.00 -166.64 -53.365

ANOVA assumes that the data are sampled from populations that follow

Gaussian distributions. This assumption is tested using the method

Kolmogorov and Smirnov:

Group KS P Value Passed normality test?

=============== ====== ======== =======================

Column A Too few values to test.

Column B Too few values to test.

Column C Too few values to test.

Intermediate calculations. ANOVA table

Source of Degrees of Sum of Mean

variation freedom squares square

============================ ========== ======== ========

Treatments (between columns) 2 151622 75811

Residuals (within columns) 6 3066.7 511.12

---------------------------- ---------- --------

Total 8 154689

F = 148.32 =(MStreatment/MSresidual)

Summary of Data

Number Standard

of Standard Error of

Group Points Mean Deviation Mean Median

=============== ====== ======== ========= ======== ========

Column A 3 1323.3 20.548 11.863 Unknown

Column B 3 1526.7 20.548 11.863 Unknown

Column C 3 1636.7 26.247 15.154 Unknown

95% Confidence Interval

Group Minimum Maximum From To

=============== ======== ======== ========== ==========

Column A Unknown Unknown 1272.3 1374.4

Column B Unknown Unknown 1475.6 1577.7

Column C Unknown Unknown 1571.5 1701.9

* * *

**Test AUC Brain**

One-way Analysis of Variance (ANOVA)

The P value is < 0.0001, considered extremely significant.

Variation among column means is significantly greater than expected by chance.

Tukey-Kramer Multiple Comparisons Test

If the value of q is greater than 4.339 then the P value is less than 0.05.

Mean

Comparison Difference q P value

================================== ========== ======= ===========

Column A vs Column B -3499.6 325.31 *** P<0.001

Column A vs Column C -5192.8 482.70 *** P<0.001

Column B vs Column C -1693.2 157.40 *** P<0.001

Mean 95% Confidence Interval

Difference Difference From To

================================== ========== ======= =======

Column A - Column B -3499.6 -3546.3 -3452.9

Column A - Column C -5192.8 -5239.5 -5146.2

Column B - Column C -1693.2 -1739.9 -1646.6

ANOVA assumes that the data are sampled from populations that follow

Gaussian distributions. This assumption is tested using the method

Kolmogorov and Smirnov:

Group KS P Value Passed normality test?

=============== ====== ======== =======================

Column A Too few values to test.

Column B Too few values to test.

Column C Too few values to test.

Intermediate calculations. ANOVA table

Source of Degrees of Sum of Mean

variation freedom squares square

============================ ========== ======== ========

Treatments (between columns) 2 4.208E+07 2.104E+07

Residuals (within columns) 6 2083.1 347.19

---------------------------- ---------- --------

Total 8 4.208E+07

F = 60600 =(MStreatment/MSresidual)

Summary of Data

Number Standard

of Standard Error of

Group Points Mean Deviation Mean Median

=============== ====== ======== ========= ======== ========

Column A 3 22334 14.408 8.318 Unknown

Column B 3 25833 23.347 13.479 Unknown

Column C 3 27527 16.997 9.813 Unknown

95% Confidence Interval

Group Minimum Maximum From To

=============== ======== ======== ========== ==========

Column A Unknown Unknown 22298 22370

Column B Unknown Unknown 25775 25891

Column C Unknown Unknown 27484 27569

* * *
